# Biomechanical Analysis of Fixation Strength in Unstable Intertrochanteric Femoral Fracture Models Based on the Caput–Collum–Diaphyseal Angle of Cephalomedullary Nails and Position of Lag Screws

**DOI:** 10.3390/jcm14186495

**Published:** 2025-09-15

**Authors:** Yong-Cheol Yoon, Sung-Jae Lee, Hyung Keun Song

**Affiliations:** 1 Orthopedic Trauma Division, Trauma Center, Gachon University College of Medicine, Namdong-gu, Incheon 21565, Republic of Korea; dryoonyc@gmail.com; 2 Department of Biomedical Engineering, Inje University, Gimhae 50834, Republic of Korea; biomech100@gmail.com; 3 Department of Orthopaedic Surgery, Ajou University School of Medicine, Yeongtong-gu, Suwon 16499, Republic of Korea

**Keywords:** hip fractures, intramedullary nails, biomechanical phenomena, caput–collum–diaphyseal angle, lag screw position

## Abstract

**Background/Objectives:** The combined effect of femoral neck–shaft angle and lag screw position on unstable intertrochanteric fracture fixation has not been well established. This biomechanical study evaluated the effects of two caput–collum–diaphyseal (CCD) angles and two lag screw positions on construct stability. **Methods:** Twenty-four synthetic femurs with identical AO/OTA 31-A2.2 fracture gaps (2 mm) were fixed using cephalomedullary nails with CCD angles of either 125° or 130°, each with a central or inferior (calcar) lag screw (*n* = 6/group). Constructs were tested in a single-leg stance under preloading, cyclic loading (75–750 N, 10,000 cycles, and 2 Hz), and axial loading to failure. Lag screw migration was measured radiographically, and femoral head rotation was recorded using a three-dimensional coordinate-measuring device. Stiffness, failure load, and rotations were compared using the Kruskal–Wallis and Bonferroni post hoc tests. **Results:** The 125° inferior configuration showed the highest stiffness (188 ± 15 N/mm, *p* = 0.038) and failure load (1350 ± 97 N, *p* = 0.047), with the least screw migration (0.54 ± 0.11 mm, *p* = 0.003), significantly outperforming the 125° central and 130° central constructs. However, it exhibited greater varus collapse (2.25 ± 0.27°, *p* = 0.013) and axial rotation (~20–30% higher than others, *p* = 0.025). Screw position had a stronger effect on stability than the CCD angle, although the 130° inferior construct showed slightly less varus deformation. **Conclusions:** An inferior calcar-guided lag screw improves fixation strength and stiffness in unstable intertrochanteric fractures, particularly in those with a 125° nail. However, this configuration increases varus and rotational displacement, warranting adjunct measures to enhance rotational control in clinical applications.

## 1. Introduction

Hip fractures are a significant health concern among older individuals. According to the International Osteoporosis Foundation, approximately 1.6 million hip-related fractures occur each year worldwide, with projections increasing to 4.5 million by the year 2050 [1]. Approximately 25% of patients with hip fractures die within a year, and 50% of survivors experience a decline in mobility and difficulties in daily living [2]. Such fractures pose serious personal and socioeconomic challenges. The primary goal of treating hip fractures is to restore patient mobility and activity levels as quickly as possible, thereby reducing complications from prolonged bed rest (e.g., pressure ulcers, urinary infections, joint contractures, pneumonia, deep vein thrombosis, pulmonary embolism, and deconditioning of cardiopulmonary function) [3,4].

Early surgical fixation is the mainstay of treatment for hip fractures. However, surgical decision-making can be complicated by advanced age, poor bone quality (osteoporosis), and medical comorbidities of patients [5]. Unstable intertrochanteric femoral fractures, often associated with osteoporotic bone and comminuted fracture patterns, present a particular challenge, with reported fixation failure and instability rates of 8–50% [6]. Early mobilization after fixation is desirable, even in unstable fractures, and cephalomedullary nails are generally recommended to provide sufficient stability for early weightbearing [7].

Outcomes of unstable intertrochanteric fracture fixation depend on numerous factors, including patient characteristics, fracture morphology, and surgical technique. Importantly, several surgical variables are under the surgeon’s control, including implant type, nail length and diameter, number of distal locking screws, and lag screw position within the femoral head [8]. A key technical metric is the tip–apex distance (TAD), which is the radiographic distance from the tip of the lag screw to the apex of the femoral head and is typically minimized (the screw is placed centrally on both anteroposterior (AP) and lateral views) to reduce the risk of cut-out. Recent modifications of this concept, such as the calcar-referenced TAD (Cal-TAD), suggest that placing the lag screw closer to the inferomedial cortical bone (calcar) of the femoral neck confers greater purchase of dense bone and improves stability [9,10]. Additionally, modern cephalomedullary nails commonly have a caput–collum–diaphyseal (CCD) angle of 125° or 130° to match the patient’s native neck angle and fracture reduction [11]. The choice of a CCD angle of 125° vs. 130° could affect lag screw positioning within the femoral head and overall alignment of the fixation. Surprisingly, the interplay between the CCD angle of the nail and lag screw position has not been biomechanically quantified in previous studies [12]. In particular, some have hypothesized that using a nail with a larger CCD angle (e.g., 130°) or achieving a slight valgus reduction can help prevent varus collapse and implant failure; however, robust biomechanical evidence is lacking [13].

This study was designed to address the lack of robust biomechanical evidence by systematically evaluating, in a controlled unstable intertrochanteric fracture model, how different nail CCD angles (125° vs. 130°) and lag screw positions (central vs. inferior) jointly influence fixation stability. We used a novel experimental design with computer numerical control (CNC)-machined synthetic bone fractures to ensure that each specimen had an identical unstable fracture pattern, thereby enhancing the reproducibility of comparisons [14]. Additionally, we utilized a three-dimensional (3D) coordinate measurement system (MicroScribe^®^) to track femoral head fragment rotations in three planes, an aspect often missed by traditional two-dimensional radiographic analyses [15]. Recent work by Moldovan et al. further demonstrated the utility of 3D technologies by introducing a structured integration and alignment algorithm to optimize surgical treatment strategies for tibial plateau fractures [16]. By leveraging these advanced techniques, this study built on prior biomechanical research and aimed to provide new insights into how implant design (CCD angle) and screw placement affect fixation strength and failure modes. We hypothesized that inferior placement of the lag screw would enhance fixation strength and stiffness compared with central placement and that the CCD angle (125° vs. 130°) would have a secondary, modulating effect on construct stability.

## 2. Materials and Methods

### 2.1. Specimen Preparation

To ensure consistency in biomechanical properties, this study used standardized synthetic bone models instead of cadaveric femora, which can vary significantly in bone density, geometry, and fracture morphology. Twenty-four identical synthetic proximal femur models (model LD2220, SYNBONE AG, Malans, Switzerland) were used, and their dimensions and mechanical properties are summarized in Table 1 [17]. The implants chosen for fixation were Zimmer Natural Nail (ZNN) cephalomedullary femoral nails (Zimmer, Warsaw, IN, USA), which matched the synthetic femur dimensions (Table 2). Two CCD angles of the nails were tested (125° and 130°), as described below.

### 2.2. Fixation Device Insertion

**Figure 1:**
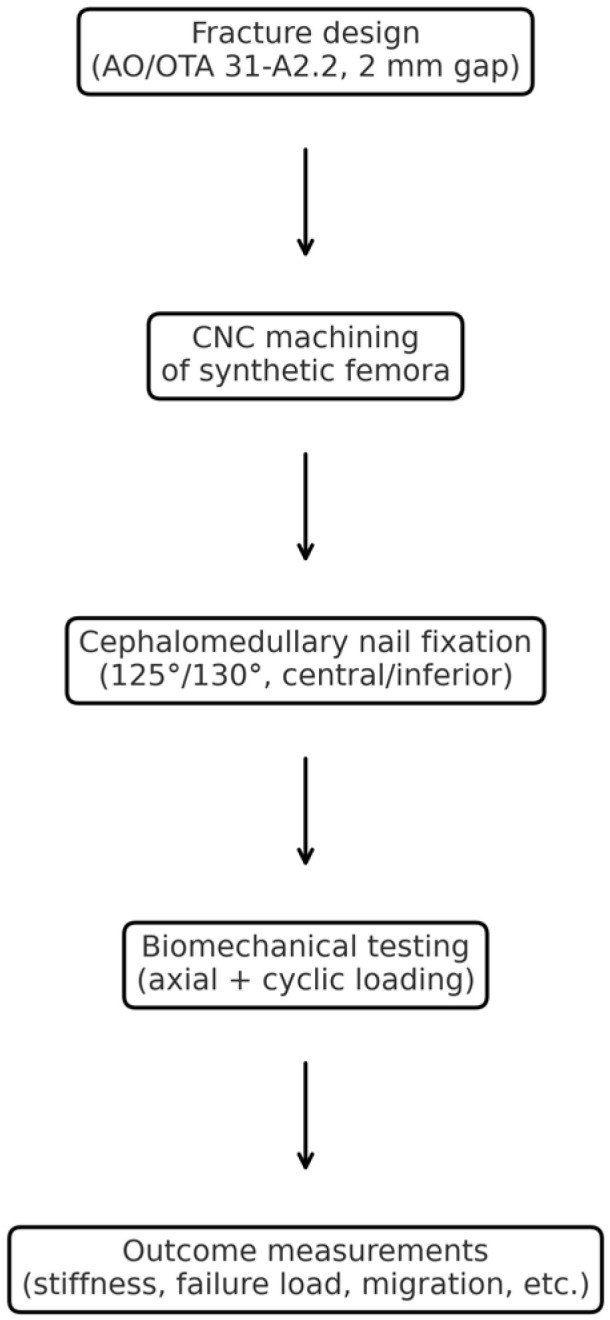
Workflow of the experimental process, including fracture design, CNC machining, nail fixation, biomechanical testing, and outcome measurements.

**Figure 2:**
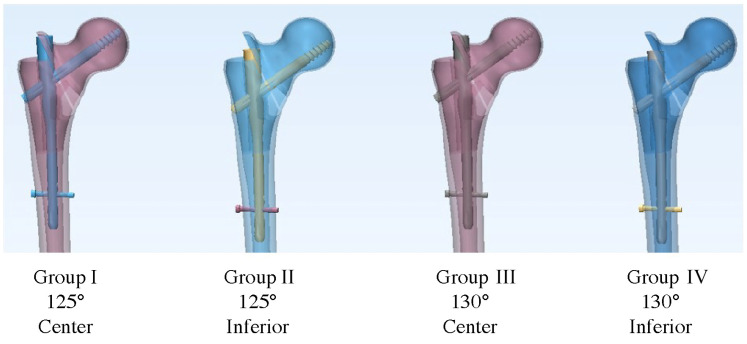
Configuration of intramedullary nail fixation models. Four groups were tested: group I, 125° nail with centrally placed lag screw; group II, 125° nail with inferior (calcar-guided) lag screw; group III, 130° nail with central lag screw; and group IV, 130° nail with inferior lag screw.

For consistent implant positioning, intramedullary nails were inserted into the femoral models before creating the fracture to ensure uniform starting alignment across specimens (Figure 1) [18,19]. Group I comprised models fixed with a 125° CCD angle nail and lag screw placed at the center of the femoral neck (center–center position). In group II, a 125° CCD angle nail was used with a lag screw positioned inferiorly (i.e., toward the calcar in the lower half of the neck on AP view). In group III, a 130° CCD angle nail with a centrally placed lag screw was used, while in group IV, a 130° nail with an inferiorly positioned lag screw was used. These configurations are illustrated in Figure 2. Each group comprised six specimens (*n* = 6). All nail insertions were performed by a single orthopedic surgeon through the tip of the greater trochanter following the standard technique. To ensure precise and reproducible lag screw placement, we adapted the technique described by Kwak et al. using a posterior cruciate ligament tibial guide as an external guide for the lag screw guide pin [20]. Each nail was locked distally with a single 5 × 40 mm screw, and a 100 mm length lag screw (10.5 mm diameter) was used proximally. Correct implant positioning was confirmed via fluoroscopy, and TAD was maintained below 25 mm in all cases to ensure a comparably acceptable lag screw depth.

### 2.3. Fracture Model Creation

**Figure 3:**
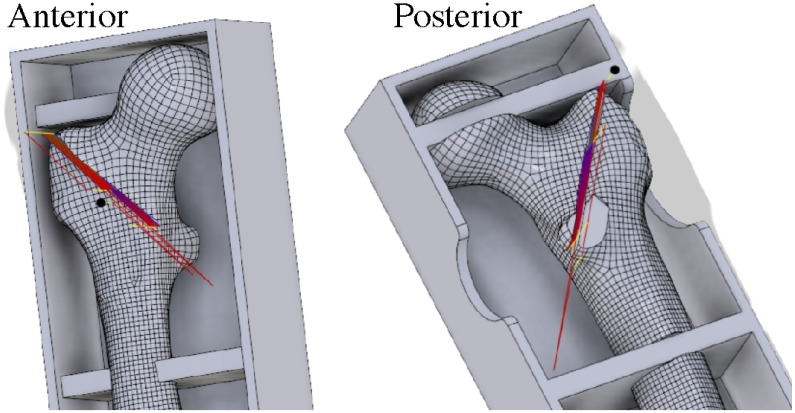
Computer-assisted design of fracture line. Blueprint of the AO/OTA 31-A2.2 unstable intertrochanteric fracture (2 mm gap with posteromedial fragment) created using three-dimensional scanning and computer numerical control (CNC) modeling.

**Figure 4:**
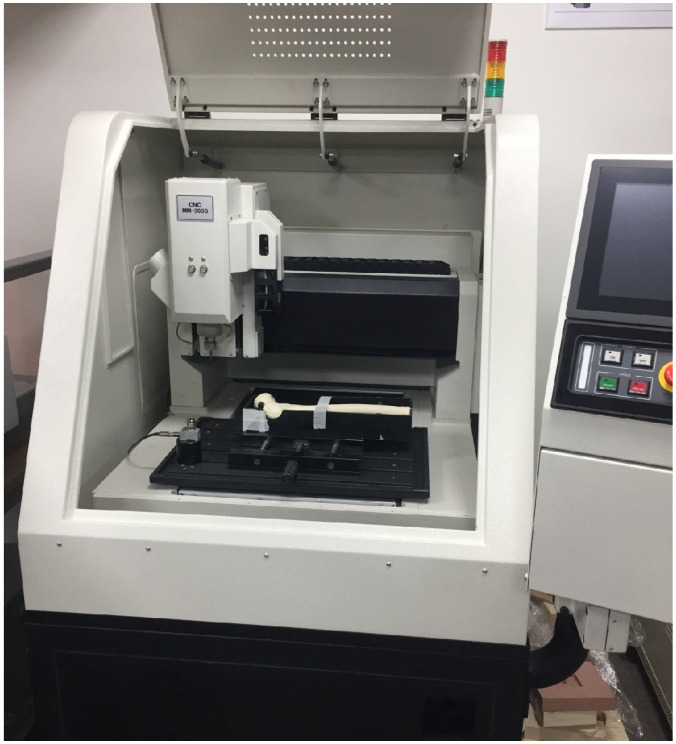
CNC milling setup. Photographs of the computer numerical control (CNC) engraving machine were used to machine identical unstable fracture lines into the synthetic femora according to the digital blueprint.

**Figure 5:**
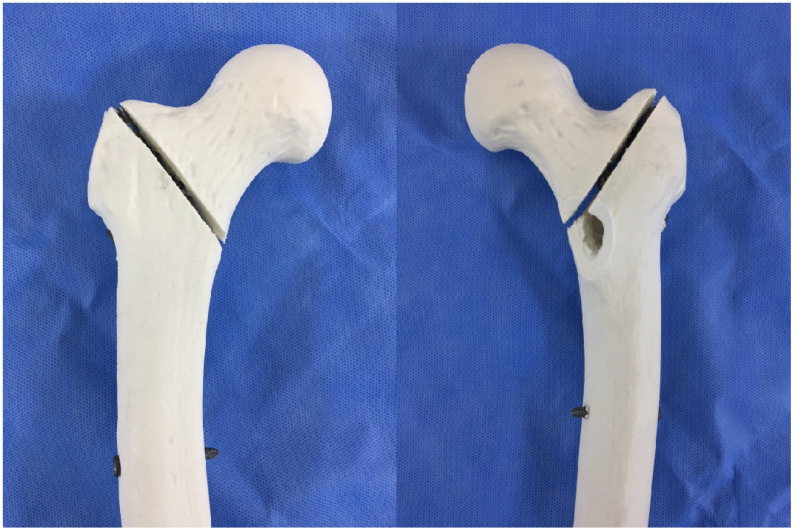
Representative unstable intertrochanteric fracture model (AO/OTA 31-A2.2). A synthetic femur with a standardized fracture, including a 2 mm fracture gap and a posteromedial fragment, is shown fixed with a cephalomedullary nail prior to biomechanical loading.

The fractures in all the specimens simulated an unstable intertrochanteric pattern (AO/OTA classification 31-A2.2). To create identical fracture geometry in each synthetic femur, a high-resolution 3D scanner (HandySCAN 700™, Creaform, Lévis, QC, Canada) was first used to digitize an intact femur model and design a reproducible fracture pattern (vertical fracture with posteromedial fragment) with a 2 mm gap [21,22]. This fracture blueprint was then programmed into a computer numerical control (CNC) milling machine (MM-300S, MANIX, Seoul, Republic of Korea), which precisely machined the fracture line and removed a 2 mm bone segment to simulate the fracture gap (Figure 3) [23]. This process yielded 24 specimens with virtually identical fracture morphologies (see Figure 4 and Figure 5 for examples of the created fracture gaps and fragment configurations; in particular, Figure 5 illustrates the standardized AO/OTA 31-A2.2 fracture with a 2 mm gap and posteromedial fragment fixed with a cephalomedullary nail prior to biomechanical testing).

### 2.4. Biomechanical Testing

Each instrumented femur was placed in industrial resin at the distal end and mounted on a testing frame in a one-legged stance orientation. The model was positioned at 25° adduction in the coronal plane (to mimic physiological hip offset during a single-leg stance) and aligned vertically in the sagittal plane [24]. An MTS 858 Mini Bionix^®^ servohydraulic material testing machine (MTS Systems Corp., Eden Prairie, MN, USA) was used to apply loads in three phases: preload, cyclic loading, and quasi-static load to failure [25,26]. Preloading was applied to ensure contact between the femoral head and the loading jig, gradually reaching 100 N at a rate of 20 N/min [27]. Dynamic loading was then performed to simulate walking with loads oscillating between 75 and 750 N at 2 Hz for 10,000 cycles. This frequency corresponds to the physiologic loading rate of normal walking, as reported in in vivo hip contact force analyses [28], and the total number of cycles represents an accelerated simulation of repeated gait cycles frequently used in biomechanical studies to approximate early postoperative functional loading. Finally, quasi-static loading to failure was conducted by increasing the axial load to 10 mm/min until either catastrophic construct failure or a 10 mm displacement of the actuator was achieved. Failure was defined as the point at which a sharp drop in load was observed concomitant with gross deformation or when the 10 mm displacement endpoint was reached.

### 2.5. Measurement of 3D Fragment Rotation

**Figure 6:**
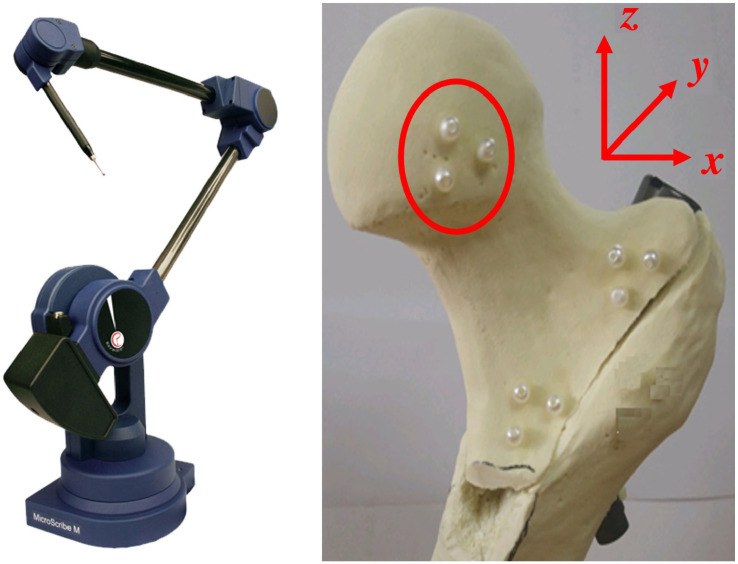
Measurement system and marker placement. *Microscribe*^®^ *M* portable coordinate measurement device (Revware Inc., Raleigh, NC, USA) with MicroScribe Utility Software, Version 7.1, and proximal femur specimen with radiopaque beads attached to the femoral head to track three-dimensional fragment movements. The red circle highlights the bead markers attached to the femoral head.

**Figure 7:**
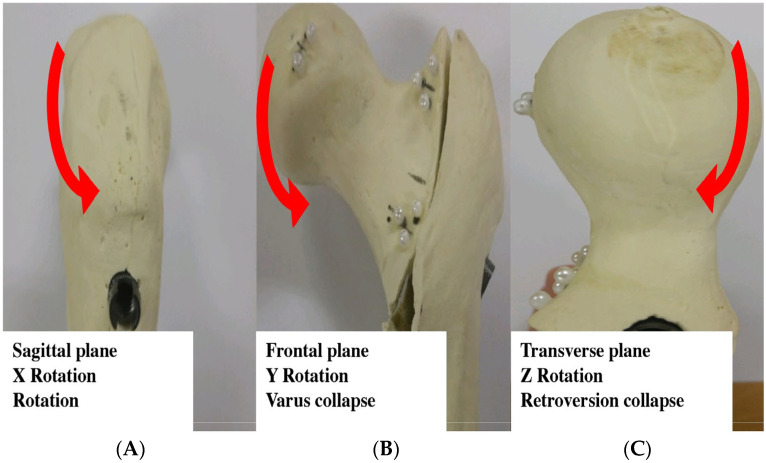
Definition of deformity directions. Illustrations of proximal femoral head–neck fragment motions: (**A**) sagittal plane rotation (x-axis), (**B**) frontal plane varus collapse (y-axis), and (**C**) transverse plane retroversion (z-axis).

To quantify movement of the proximal fracture fragment (femoral head and neck) in 3D, we used a Microscribe^®^ M (Revware Inc., Raleigh, NC, USA) portable coordinate measurement system [23]. Before testing, three 1 mm steel marker beads were affixed to the femoral head of each specimen. The 3D coordinates of these markers were recorded at different testing stages and later converted into rotations about anatomical axes. Specifically, the marker coordinates were measured after preloading, cyclic loading, and failure tests. The recorded coordinates were processed using the MicroScribe Utility Software, Version 7.0 (Revware Inc., Raleigh, NC, USA) to compute rotations about the three principal axes (defined relative to the femur): (1) rotation about the x-axis (sagittal plane rotation of the head fragment, corresponding to flexion/extension or anterior/posterior tilt of the head); (2) rotation about the y-axis (coronal plane rotation, corresponding to varus–valgus collapse of the head/neck fragment); and (3) rotation about the z-axis (axial rotation, corresponding to changes in femoral neck anteversion/retroversion). The rotation angles were calculated using Bryant angles and rotation matrices, considering the post-preload configuration as the baseline (zero reference) for each specimen [29]. Each measurement was performed thrice for repeatability, and the average values of rotational change after dynamic loading were used for the analysis (Figure 6 illustrates the marker placement and coordinate system, and Figure 7 demonstrates the rotation calculation method).

### 2.6. Measurement of Lag Screw Migration

Anteroposterior radiographs of each construct were obtained immediately after insertion (preload) and after the completion of all mechanical loading. Using a computer-aided design software (Rhinoceros^®^ 3D, version 7, Robert McNeel & Associates, Seattle, WA, USA), we measured the lag screw displacement relative to the surrounding bone between the two time points. This quantified the amount of “lag screw sliding” or migration that occurred during loading [30,31]. Measurements were taken along the femoral neck axis on the radiographs, with positive values indicating that the screw moved (backed out) relative to the bone.

### 2.7. Statistical Analysis

All data were analyzed using SPSS Statistics (v20.0, IBM Corp., Armonk, NY, USA). Given the sample size and data distribution, a non-parametric Kruskal–Wallis test was used to compare the four groups for each outcome measure (rotational displacement, screw migration, stiffness, and failure load). When a significant overall difference was found (*p* < 0.05), pairwise post hoc comparisons were performed with Bonferroni correction to identify the specific groups that differed. Additionally, we examined the magnitude of differences (mean differences and percentages) to assess effect size relevance on key outcomes. Statistical significance was set at *p* < 0.05 for all tests (after adjustment for multiple comparisons where applicable).

## 3. Results

### 3.1. Three-Dimensional Femoral Head Rotational Deformation

After dynamic loading, all the specimens showed rotational displacement of the proximal fragment in each plane. Overall, group II (125° nail with inferior lag screw) exhibited the largest rotation in all axes. Maximum rotation about the x-axis (sagittal plane “head rotation”) was observed in group II at 1.72 ± 0.09°, whereas the other groups showed smaller values (group I: 1.42 ± 0.19°; group III: 1.33 ± 0.17°; group IV: 1.66 ± 0.18°) (Table 3). Post hoc comparisons (Bonferroni-corrected) indicated that the x-axis rotation in group II was significantly greater than that of groups I (*p* = 0.010) and III (*p* = 0.001). The rotation of group IV (1.66°) was the second highest and was not significantly different from that of group II (*p* > 0.05), reflecting a similar trend when the lag screw was placed inferiorly despite the different CCD angle.

For rotation about the y-axis (varus collapse in the coronal plane), group II again showed the greatest change, at 2.25 ± 0.27°. The other groups had markedly lower varus deformation (group I: 1.94 ± 0.41°; group III: 1.94 ± 0.25°; group IV: 1.97 ± 0.44°). The varus collapse in group II was significantly larger than that in groups I (*p* = 0.031), III (*p* = 0.0025), and IV (*p* = 0.015) after the Bonferroni correction. In contrast, the differences among groups I, III, and IV were small (all approximately 1.9° of varus collapse) and not statistically significant. These findings indicate that the inferior lag screw with a 125° nail (group II) experienced approximately 15% more varus collapse than the other configurations under identical loading.

Rotation about the z-axis (changes in femoral head anteversion/retroversion) showed a trend toward higher values in the groups with inferior screw placement (group II: 0.58 ± 0.21°; group IV: 0.58 ± 0.14°) compared with the central screw groups (group I: 0.51 ± 0.12°; group III: 0.49 ± 0.15°). However, variation in z-axis rotation did not reach statistical significance among the four groups (overall, *p* = 0.280). This suggests that, unlike varus collapse, the effect of the different configurations on femoral head torsional rotation (internal/external rotation) was minor and within experimental variability.

### 3.2. Lag Screw Migration

Differences were observed in the amount of lag screw sliding (migration) between the groups. Group I (125° central) showed the greatest screw migration during testing, with the lag screw backing out by 0.64 ± 0.10 mm on average. Group II (125° inferior) had the least migration at only 0.54 ± 0.11 mm. The 130° nail groups were intermediate, with group III (130° central) showing 0.62 ± 0.11 mm migration and group IV (130° inferior) showing 0.56 ± 0.07 mm. Statistical analysis confirmed a significant overall group effect (*p* = 0.003) (Table 3). Post hoc tests revealed that screw migration in group II was significantly lower than that in groups I (*p* = 0.038) and III (*p* = 0.027). There was no significant difference between groups II and IV (*p* > 0.05), suggesting that the inferior lag screw position minimized sliding regardless of the nail angle. No significant difference was found between groups I and III (central screw, different CCD angles) or between groups III and IV, indicating that screw position had a more pronounced effect on migration than nail angle. Placing the screw inferiorly (group II vs. group I) reduced lag screw sliding by approximately 16% under these test conditions.

### 3.3. Construct Stiffness and Failure Load

**Figure 8:**
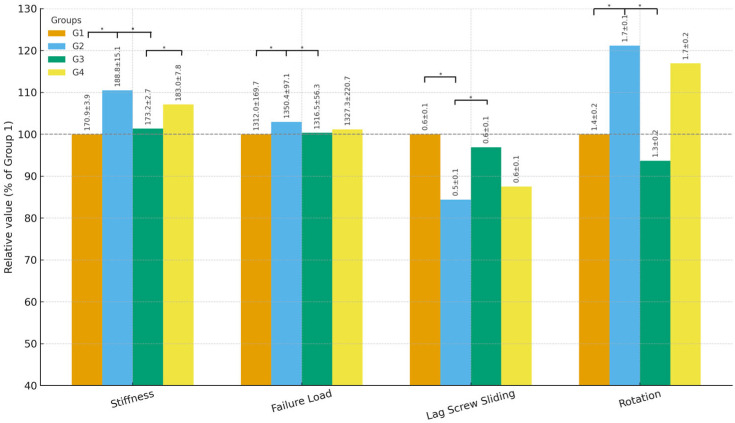
Relative biomechanical performance metrics normalized to group 1 (125° central). Bar graphs illustrate stiffness, failure load, lag screw sliding, and femoral head rotation, with mean ± SD values indicated on the bars. For additional outcomes, including varus collapse and retroversion, please refer to Table 3. (* *p* < 0.05, Bonferroni-corrected).

The stiffness (axial load–displacement response) and ultimate failure load of each construct were measured during the static loading phase. Group II demonstrated the highest stiffness at 188.8 ± 15.1 N/mm, whereas group I was the least stiff at 170.9 ± 3.9 N/mm. Groups III and IV showed intermediate stiffness values (group III: 173.2 ± 2.7 N/mm; group IV: 183.0 ± 7.8 N/mm). Thus, the 125° inferior configuration (group II) was approximately 10.5% stiffer than the 125° central configuration (group I) and approximately 9% stiffer than the 130° central configuration (group III). Statistical analysis confirmed a significant difference between the groups (*p* = 0.038) (Table 3). Post hoc comparisons indicated that the stiffness in group II was significantly greater than that in groups I (*p* < 0.001) and III (*p* < 0.001). There was no significant difference in stiffness between groups II and IV (*p* > 0.05), reflecting a similar advantage conferred by the inferior screw position in the 125° and 130° nails. Group IV (130° inferior) was also significantly stiffer than group III (130° central, *p* < 0.01). No difference was detected between groups I and III, suggesting that changing the CCD angle from 125° to 130° with the screw in the center did not appreciably affect the construct stiffness in this model (Figure 8).

The failure load (maximum load sustained) exhibits a similar pattern. Group II had the highest mean failure load at 1350.4 ± 97.1 N. Group I had the lowest failure load at 1312.0 ± 169.7 N. Groups III and IV had mean failure loads of 1316.5 ± 56.3 N and 1327.3 ± 220.7 N, respectively. Although the absolute differences in failure load between the configurations were relatively small (in the order of 30–40 N, or a few percent of the total load), the overall comparison was statistically significant (*p* = 0.047). Post hoc tests showed that the failure load of group II was significantly higher than that of groups I (*p* < 0.001) and III (*p* < 0.001). Regarding failure load, there was no significant difference between groups II and IV (*p* > 0.05) or between groups I and III. In practical terms, the 125° inferior configuration withstood an approximately 3% higher load before failure than the 125° central configuration, whereas the 130° configuration fell in between. All the constructs failed due to varus bending collapse of the fracture, accompanied by lag screw cut-out or proximal nail deformity rather than distal nail breakage.

## 4. Discussion

Successful fixation of unstable intertrochanteric femoral fractures requires careful consideration of multiple factors. Patient-related (age, bone mineral density, and general health), fracture-related (fracture pattern, comminution, and reduction quality), and surgical (implant selection and placement technique) variables all contributed to outcomes. Although patient and fracture characteristics are mostly predetermined and not modifiable by the surgeon, surgical techniques and implant choices can be adjusted. In this study, we controlled for bone quality (using identical synthetic bones), fracture type (using the same AO 31-A2.2 fracture pattern in all specimens), and reduction (consistent anatomic alignment achieved by the preset jigs) to isolate the effects of two modifiable surgical factors: the nail’s CCD angle and lag screw’s position within the femoral head. Thus, we aimed to provide data to guide surgeons in choosing the optimal construct for a given patient or fracture scenario.

This investigation offers several novel insights owing to its experimental design. Unlike previous biomechanical studies, which often evaluated different implants or screw positions in isolation, our study simultaneously examined the influence of the lag screw position and nail neck–shaft angle using a rigorously standardized model [20,32]. The use of CNC-machined synthetic bone fractures ensured a highly reproducible fracture geometry and comparable test conditions across all samples. Furthermore, by tracking 3D rotation of the femoral head segment using a coordinate measurement device, we captured subtle rotational instabilities that plain radiographs or two-dimensional measurements might miss. These methodological advances allowed us to clearly distinguish this work from previous studies and provide new data on how implant design and placement interact to affect fixation strength.

Our findings confirm and extend the observations of earlier studies. Kuzyk et al. previously reported that inferior placement of the lag screw (closer to the calcar in the AP view) significantly increased the axial stiffness and reduced the risk of cut-out compared with a centrally or superiorly placed screw [33]. The results of the current study strongly support this finding; in the 125° and 130° nail groups, the inferior (calcar-guided) lag screw position (groups II and IV) produced higher stiffness and less screw migration than the respective central screw position (groups I and III). The inferior lag screw engages the dense bone above the calcar femorale, thereby better resisting compression and translation. This aligns with the concept of Cal-TAD and offers greater stability. Additionally, we found that the constructs with inferior screw placement had slightly higher failure loads (although differences in the ultimate load were modest), indicating that the initial stiffness and resistance to sliding translated into marginally improved load-bearing capacity.

A clinically relevant question is whether using a nail with a smaller CCD angle (e.g., 125°) or a larger angle (130°) affects the likelihood of varus collapse. Some authors have advocated valgus reduction and larger CCD angle nails to reduce varus stress on the implant, noting that varus malreduction is associated with a higher risk of lag screw cut-out [34,35]. In theory, a nail with a larger CCD angle (130°) allows the surgeon to maintain a greater valgus or anatomic neck angle during fixation, potentially decreasing the tendency for the head–neck fragment to settle into varus. Our results provide biomechanical evidence to support this concept; the 125° nail with an inferior screw (group II) experienced significantly more varus collapse (approximately 0.3° more or ~15% higher) than the 130° nail with an inferior screw (group IV) under identical loads. In other words, when the lag screw was placed in the same inferior position, the smaller-angle nail allowed the proximal fragment to tip into the varus more readily than the larger-angle nail. We suspect that this is because the 125° nail, by design, results in a slightly more varus starting alignment (or requires a varus reduction to fit the patient’s anatomy if the native neck angle is ~130°), meaning that there is less residual capacity to accommodate further varus deformation before the construct starts to fail. This observation fills an important gap in the literature—previously, the influence of nail CCD angle on varus stability had not been well quantified, as prior biomechanical studies focused mainly on different implant types (e.g., sliding hip screw vs. nail or on screw position alone). Our data suggest that in cases prone to varus collapse, opting for a larger CCD angle nail may indeed provide a slight biomechanical advantage in maintaining alignment [20,21,36].

Notably, when comparing groups with similar lag screw positions (e.g., group I vs. group III, both center-to-center; group II vs. group IV, both inferior), the differences in their biomechanical performance were relatively small. Groups I (125° central) and III (130° central) had very similar stiffness, varus collapse, and screw migration, indicating that simply changing the nail angle without changing the screw position did not markedly affect the stability in our model. Similarly, groups II (125° inferior) and IV (130° inferior) were comparable in many metrics (apart from the slight varus difference mentioned above). These findings underscore that lag screw placement (central vs. inferior) was the dominant factor affecting construct stability, whereas the nail neck angle played a secondary role. This aligns with the general principle behind the TAD: placing the lag screw in the dense subchondral bone near the center of the femoral head (or slightly inferior in the AP view) is critical for preventing excessive collapse and cut-out [9]. Our results reinforce that achieving a low TAD or Cal-TAD by positioning the screw in the “center-center” or “lower-center” region is more important than small differences in nail geometry, provided the fracture is well reduced [34,37].

Despite the superior overall performance of the 125°inferior configuration (group II) in terms of load bearing and stiffness, a tradeoff was observed in the form of increased rotational instability [38]. Group II showed the highest tendency for the femoral head/neck fragment to rotate (twist) and collapse into varus under load. This rotational movement can be clinically deleterious as it may contribute to lag screw cut-out if the femoral head rotates into a varus and retroverted position, levering the screw upward and out of the head. In practical terms, our biomechanical data suggest that while a 125° nail with an inferior screw offers robust resistance to vertical load (thus allowing earlier weight-bearing or use in heavier patients), it should be used with caution in osteoporotic bone or highly unstable fractures because it may permit more varus and torsional displacement [39]. These additional movements could jeopardize fixation if not controlled. In contrast, the 130° nail with a central or inferior screw tended to allow slightly less varus collapse (and group IV had a slightly lower, although not statistically different, rotational change than group II), which might make the 130° configuration a safer choice in patients at a high risk of collapse (e.g., very low bone density or medial wall deficiency). This insight is consistent with recent clinical findings that emphasize the importance of restoring an appropriate neck–shaft angle during surgery; poor (varus) reduction cannot be fully compensated for by an optimal screw position alone.

Clinically, these findings can inform surgical decision-making regarding intertrochanteric fractures. For example, in a younger patient or one with relatively good bone quality who needs immediate weight-bearing, a 125° nail with an inferiorly placed lag screw (calcar support) may be chosen to maximize fixation strength and stiffness. In such cases, the surgeon gains the benefit of higher construct rigidity (approximately 10% higher stiffness in our tests), which could improve the stability under a load. Conversely, in an older patient or a fracture with a large medial cortical defect, the same 125° inferior construct might carry a higher risk of varus collapse and screw cut-out, owing to its observed tendency for greater varus and rotational movement. In these scenarios, a 130° nail (to facilitate greater valgus or anatomical reduction) or a more centrally placed lag screw may be preferred to avoid excessive varus deformation. In other words, a slightly more flexible construct (130° with a central screw) that maintains alignment might ultimately be safer for very weak bones, even if its immediate stiffness is slightly lower, because preventing catastrophic failure (cut-out) is a priority. Therefore, surgeons should tailor the nail selection and screw position to the patient’s bone quality and fracture geometry, balancing the goal of mechanical stability against the risk of collapse. Our results also suggest that if a 125° inferior construct is used (owing to its superior initial fixation strength), one should be proactive in counteracting its varus tendency. Achieving a near-anatomical or slight valgus reduction of the fracture is an important step. Additional intraoperative strategies include the use of blocking screws or a trochanteric stabilization plate to support the medial cortex, augmenting fixation with bone cement, or a second anchor (as in a dual-screw or blade design) to improve rotational control [40]. These measures, combined with the inherently stronger 125° inferior configuration, may help mitigate the increased varus and rotational movements, leading to a more favorable outcome.

Another notable aspect of our study is the relationship between varus collapse and rotational instability. The configuration that allowed the greatest varus deformation (group II) also exhibited the greatest femoral head rotation about the longitudinal axis. This suggests a coupled failure mode; as the proximal fragment collapses into varus, it may also rotate (externally or internally), owing to asymmetric loading and lack of constraints, exacerbating the risk of fixation failure. Although our sample size was limited, future analyses with larger datasets should examine the correlation between varus collapse angles and rotational displacements, as well as between these displacements and overall construct stiffness. A better understanding of these correlations could help predict failure. For example, one might find that beyond a certain degree of varus settling, the risk of a screw beginning to cut out increases sharply, especially if accompanied by rotational instability. Identifying such thresholds would be valuable for surgeons during postoperative monitoring (e.g., if early radiographs show varus settling, it might prompt closer observation or protective weight-bearing to prevent further rotation and cut-out) [41,42,43]. In our data, we qualitatively observed that specimens with minimal varus maintained better overall stiffness, whereas those with larger varus collapse had more rotation and tended to fail at slightly lower loads; however, a formal statistical correlation was not performed. We recommend that future biomechanical studies and clinical investigations should assess these relationships; for example, measuring whether patients with varus progression on radiograph also show increased femoral head rotation and higher failure rates. Such insights will bridge the gap between laboratory findings and clinical outcomes [42].

This study has several limitations. First, uniform synthetic bone models were used instead of cadaveric femora. While they ensured consistency across specimens, they do not fully replicate the heterogeneity of human bone. Second, the loading protocol simulated a simplified axial single-leg stance and did not include multidirectional or torsional forces, which may affect construct behavior in vivo. Third, the relatively small sample size (six per group) limited the statistical power to detect subtle differences. Fourth, only one unstable fracture pattern (AO/OTA 31-A2.2) was tested, and the findings may not fully apply to other morphologies, such as reverse obliquity or subtrochanteric extension. Finally, this purely biomechanical study did not account for biological healing or remodeling, which are critical in clinical outcomes. Despite these limitations, our results provide clinically relevant guidance: a 125° nail with an inferior lag screw enhances fixation strength but also increases varus and rotational displacement, underscoring the need for careful reduction and supplementary stabilization strategies in clinical practice.

## 5. Conclusions

This biomechanical study examined how lag screw position (central vs. inferior in the femoral neck) and nail CCD angle (125° vs. 130°) affected fixation stability in an unstable intertrochanteric femur fracture model. We found that inserting the lag screw in the inferior portion of the neck (calcar region) significantly enhanced fracture fixation strength and stiffness, particularly when combined with a 125° nail. However, this configuration also resulted in greater varus collapse and rotational motion of the head–neck fragment. A 125° CCD nail with an inferior lag screw provided the highest load-bearing capacity but also showed an approximately 20–30% increase in rotational displacements compared with other configurations. Therefore, if this high-stability construct is used, surgeons should implement strategies to counteract varus and rotational deformations (e.g., ensuring proper reduction and adequate supplementary support) to optimize clinical outcomes. In summary, the lag screw position and nail geometry are important considerations; the inferior calcar screw position improves mechanical stability, and selecting a larger CCD angle nail (130°) may reduce varus propensity. The optimal construct ultimately depends on individual patient factors, and careful surgical techniques are required to balance these effects in each case.

## Figures and Tables

**Table 1:** Characteristics of the bone model.

Length	337 mm
Neck angle	135°
Anteversion	15°
Head diameter	48 mm
Canal diameter	10 mm
Material	Cortical low density Soft cancellous bone

**Table 2:** Characteristics of implants.

Nail length	180 mm
Nail diameter	10 mm
CCD angle	125°/130°
Lateral bending	4°
Anteversion	15°
Lag screw length	100 mm
Lag screw diameter	10.5 mm
Distal locking screw diameter	5 mm
Distal locking screw length	40 mm

CCD, caput–collum–diaphyseal.

**Table 3:** Biomechanical test results (mean ± SD).

Variables	Group 1	Group 2	Group 3	Group 4	*p*-Value
Stiffness (N/mm)	170.9 ± 3.9	188.8 ± 15.1	173.2 ± 2.7	183.0 ± 7.8	0.038
Failure load (N)	1312.0 ± 169.7	1350.4 ± 97.1	1316.5 ± 56.3	1327.3 ± 220.7	0.047
Lag screw sliding (mm)	0.64 ± 0.10	0.54 ± 0.11	0.62 ± 0.11	0.56 ± 0.07	0.003
Varus collapse (degree)	1.94 ± 0.41	2.25 ± 0.27	1.94 ± 0.25	1.97 ± 0.44	0.013
Rotation (degree)	1.42 ± 0.19	1.72 ± 0.09	1.33 ± 0.17	1.66 ± 0.18	0.025
Retroversion (degree)	0.51 ± 0.12	0.58 ± 0.21	0.49 ± 0.15	0.58 ± 0.14	0.280

SD, standard deviation.

## Data Availability

The datasets generated and/or analyzed during the current study are not publicly available because of restricted access to our hospital database, but they are available from the corresponding author upon reasonable request.

## Abbreviations

The following abbreviations are used in this manuscript:

TAD	Tip–apex distance
AP	Anteroposterior
CCD	Caput–collum–diaphyseal
Cal-TAD	Calcar-referenced TAD
3D	Three-dimensional
CNC	Computer numerical control
